# Response: Commentary: Statistical Modeling for the Prediction of Infectious Disease Dissemination With Special Reference to COVID-19 Spread

**DOI:** 10.3389/fpubh.2021.783201

**Published:** 2022-01-31

**Authors:** Subhash Kumar Yadav, Yusuf Akhter

**Affiliations:** ^1^Department of Statistics, School of Physical and Decision Sciences, Babasaheb Bhimrao Ambedkar University, Lucknow, India; ^2^Department of Biotechnology, School of Life Sciences, Babasaheb Bhimrao Ambedkar University, Lucknow, India

**Keywords:** distribution fitting models, time series regression models, epidemiological models of disease, parameters, estimation, prediction

With reference to the quoted commentary (1) above, commentators observed, “**In sections ‘SI and SIS Models’ and …. chance or probability.”** We would like to state that the commentators have misunderstood and misinterpreted the concept and definition of β. From the mentioned models, it may be seen that, β is the per capita per unit time infection rate. It is a disease transmission coefficient or a transmission rate as described by Kirkeby et al. (2). Bailey (3), at page no. 20, and Bailey (4), at page no. 33, have defined β as the infection rate. Chalub and Souza (5) have defined that, β may be interpreted as a rate or as a probability among many other possible choices. Jagan et al. (6) have defined β as the transmission rate; it is the number of infections per unit time per susceptible per infected. Further, Citron et al. (7) have defined as β the transmission rate, published in “*PNAS”* and others (8–20).

Further, we have mentioned that β estimates the spread rate, which shows the chance of transmission of the disease from an infectious individual to a susceptible one. Ucakan et al. (17) have explicitly expressed that the transmission coefficient, β estimates the probability of getting the disease from an infectious individual to the susceptible and stated it as a probability that ranges from 0 to 1 (0< *β* <1). On the other hand, Brauer and Castillo-Chavez (21) have defined β as the product of the probability of the transmission per contact and the per capita contact rate. In addition, Chen (22) has also defined β as the infection coefficient, the product of the average number of contacts within a given time period and the probability of infection for the contact between susceptible and infectious individuals. Thus, β may take a value >1 and, therefore, in general, it may not be considered as a probability. Furthermore, one should be very careful about the choice of β while defining diagrams for different epidemiological models. Moreover, Hethcote and Driessche (10) mentioned that the number of new cases per unit time shall be λ*SI/N*, which is called the standard incidence (23–27) with λ as a contact rate. Another common incidence is the simple mass action incidence, β*SI*, where β was defined as the transmission coefficient (4, 9). Additionally, Okabe and Shudo (16) have defined that β*SI* represents the number of susceptible individuals that get infected per day. For more details, further references can be consulted (28, 29).

In their next comment, they have wrongly pointed out **“In section, “The Distribution Fitting,” the …. biological interpretations.”** In the section “The Distribution Fitting” of our review on page no. 04, we have clearly mentioned that “it is the growth rate of infection which determines the total number of infections which depends on the numerous factors (30),” and in the context of distribution fitting, we have mentioned that the infectious disease mainly depends on two factors, namely, the number of carriers and the time of infection as reported by Datta et al. (31). They have further mentioned, **“In the section of “The Basic Reproduction Number”…… not the model.”** In the section “The Basic Reproduction Number,” the commentators have wrongly stated the concept here. From the formula, it may be observed that, *R*_0_ does not depend on time. Further, we have not mentioned *R*_0_ as a rate anywhere in the review article. It is solely their imaginary creation. We have mentioned in the section that, *R*_0_ is measured through the effective reproductive rate, denoted by *R*. Thus, we have mentioned, “effective reproductive number” *R* as “effective reproductive rate” since it depends on time (32–38). Although for minimizing the ambiguity, the use of consistent terminology throughout the literature is required, and, therefore, we appreciate the commentators. Further, commentators have wrongly mentioned that we have stated ξ as a model in the sub-section SIRS on page no. 9, however, ξ is defined in subsection SEIRS on page no. 20. In our opinion, the sentence should be started with the word “In,” which may be a typographical error, however, we have clearly mentioned that ξ is the rate by which the recovered individuals become susceptible because of the loss of immunity, and ξ is not a model. For more details, further references can be referred to (15, 39, 40).

In their last comment, they have pointed out, **“In the section, “Further Suggestions and Future Prospectives”, ….. recommendations.”** Hajian-Tilaki (41) clearly observed that in designing epidemiologic studies, sample size calculation has an important role to detect an effect and achieve the desired precision in estimates of parameters of the interest (41–45). Therefore, it is a key factor that must be considered while designing the study protocol (45). Small sample size will fail to provide a precise estimate and reliable answers to the policy makers (46). On the other hand, a large sample size than required will cause wastage of useful resources earmarked to the study (45). Malhorta and Indrayan (47) have recorded that, an adequate estimation of the correct required sample size is a must, especially in the case of such infectious diseases, for which the newly invented diagnostic tests are expensive to carry out. For any epidemiological study, the investigators must present the principles of sample size calculation to justify these numbers (44). Further, Hajian-Tilaki (48) also mentioned that, unfortunately, sample size calculations are rarely reported by clinical investigators for diagnostic studies (49, 50). The sample size calculation may be ignored wherever required, for instance, assumptions in household epidemic models for determining the transmissibility (R_0_). This can sometimes be seen based on who infected whom however, it is only applicable to the infections with a long incubation period, such as AIDS and tuberculosis. Although, for the infections with a shorter incubation period, such as Influenza and COVID-19, we must meet the sample size conditions in order to estimate the growth rate. The commentators mentioned that in the section “Further Suggestions and Future Prospective,” the compulsion of sample size determination can be avoided, however, we opined that, if certain researchers have not mentioned the assumptions regarding the sample size calculation, it does not mean that there is no need of it. Many a time, the determination of sample size is ignored, where strict ethical issues are not concerned, but it will harm in some sense or another, as mentioned by the more pragmatic scholars earlier (45, 46). Thus, it is highly recommended for an epidemiological study that the appropriate sample size calculation should be followed (41, 48, 51–58).

## Author Contributions

SY and YA have together prepared the response to the commentary. Both the authors contributed to the article and approved the submitted version.

## Conflict of Interest

The authors declare that the research was conducted in the absence of any commercial or financial relationships that could be construed as a potential conflict of interest.

## Publisher's Note

All claims expressed in this article are solely those of the authors and do not necessarily represent those of their affiliated organizations, or those of the publisher, the editors and the reviewers. Any product that may be evaluated in this article, or claim that may be made by its manufacturer, is not guaranteed or endorsed by the publisher.
